# A systematic review and meta-analysis of sexually transmitted infections and blood-borne viruses in travellers

**DOI:** 10.1093/jtm/taae038

**Published:** 2024-03-04

**Authors:** Wondimeneh Shiferaw, Beatris Mario Martin, Judith A Dean, Deborah Mills, Colleen Lau, David Paterson, Kenneth Koh, Lars Eriksson, Luis Furuya-Kanamori

**Affiliations:** UQ Centre for Clinical Research, Faculty of Medicine, The University of Queensland, Herston, Australia; Asrat Woldeyes Health Science Campus, Debre Berhan University, Ethiopia; UQ Centre for Clinical Research, Faculty of Medicine, The University of Queensland, Herston, Australia; UQ Poche Centre for Indigenous Health, Faculty of Health, and Behavioural Sciences, The University of Queensland, Toowong, Australia; UQ Centre for Clinical Research, Faculty of Medicine, The University of Queensland, Herston, Australia; Dr Deb The Travel Doctor, Travel Medicine Alliance, Brisbane, Australia; UQ Centre for Clinical Research, Faculty of Medicine, The University of Queensland, Herston, Australia; UQ Centre for Clinical Research, Faculty of Medicine, The University of Queensland, Herston, Australia; Gladstone Road Medical Centre, Brisbane, Australia; Herston Health Sciences Library, The University of Queensland, Herston, Australia; UQ Centre for Clinical Research, Faculty of Medicine, The University of Queensland, Herston, Australia

**Keywords:** Pretravel consultation, risk factor, sexual behaviour, sexual health, sexually transmitted disease, STD, tourism

## Abstract

**Background:**

Sexually transmitted infections (STIs) and blood-borne viruses (BBVs) impose a global health and economic burden. International travellers facilitate the spread of infectious diseases, including STIs. Hence, this review assessed the prevalence/proportionate morbidity of travellers with STIs and sexually transmitted BBVs and factors associated with the infection in this population.

**Methods:**

PubMed, Scopus, Web of Science, Cumulative Index to Nursing and Allied Health Literature (CINAHL), Embase and Cochrane Library were searched from inception of the databases until November 2022. Published analytical observational studies reporting the prevalence/proportionate morbidity of travellers with STIs and factors associated with STIs by type of traveller [i.e. tourists, business travellers, students, visiting friends or relatives (VFRs), international truck drivers, backpackers, expatriates and men who have sex with men (MSM)] were included. The selection of articles, data extraction and risk of bias assessment were conducted by two independent reviewers. Meta-analyses were conducted for each STI by clinical presentation and type of traveller.

**Results:**

Thirty-two studies (*n =* 387 731 travellers) were included; 19 evaluated the proportionate morbidity of STIs among symptomatic travellers, while 13 examined the prevalence of STIs in asymptomatic travellers. The highest proportionate morbidity was found among VFRs (syphilis, 1.67%; 95% CI: 1.03–2.81%), backpackers (*Chlamydia trachomatis*, 6.58%; 95% CI: 5.96–7.25%) and MSM (HIV [2.50%;95% CI: 0.44–12.88%], gonorrhoea [4.17%; 95% CI: 1.1.5–13.98%], lymphogranuloma venereum [4.17%;95% CI: 1.1.5–13.98%] and HAV [20.0%; 95% CI: 14.99–26.17%]). The highest prevalence of STIs among asymptomatic were found in MSM (HIV [25.94%; 95% CI: 22.21–30.05%] and HBV [24.90%; 95% CI: 21.23–28.96%]) and backpackers (*C. trachomatis*, 3.92%; 95% CI: 2.72–5.32%). Short duration of the trip (<1 month), not having pre-travel consultation, travelling to Southeast Asia and being unvaccinated for HBV were identified as risk factors for STIs.

**Conclusion:**

Strategies to prevent STIs and sexually transmitted BBVs should be discussed at pre-travel consultations, and recommendations should be prioritized in high-risk groups of travellers, such as backpackers, VFRs and MSMs. Additionally, healthcare providers should tailor recommendations for safe sex practices to individual travellers’ unique needs.

## Introduction

Every day, more than a million new cases of sexually transmitted infections (STIs) are acquired globally.^1^ The World Health Organization (WHO) estimates that over 374 million new infections of four common curable STIs (i.e. *Chlamydia trachomatis*, gonorrhoea, syphilis and trichomoniasis) are acquired each year.^1^ The increasing incidence of STIs and sexually transmitted blood-borne viruses (BBVs) is a significant public health concern beyond the infection itself. If left untreated, STIs may lead to a variety of complications (e.g. pelvic inflammatory disease, ectopic pregnancy, postpartum endometriosis, infertility, chronic abdominal pain in women and arthritis caused by gonorrhoea and *C. trachomatis*),^2^^,^^3^ as well as increase the risk of acquiring and transmitting human immunodeficiency virus (HIV).^4^

BBVs are a group of viruses mainly transmitted through exposure to infected blood or other body fluids. HIV, hepatitis B virus (HBV) and hepatitis C virus (HCV) are three of the most common BBVs globally. HBV is a global public health concern. In 2019, the WHO estimated that over 296 million people were living with chronic HBV infection worldwide.^5^ Recent evidence has highlighted an increasing prevalence of HBV being transmitted sexually (i.e. in heterosexuals and male homosexuals).^6^ The risk of infection may increase in regions with a chronic prevalence of HBV infection of over 2%, such as Africa and the Western Pacific.^7^ Consequently, travellers (e.g. expatriates, tourists, missionaries and aid workers) who engage in sexual activity with potentially infected partners [i.e. hepatitis B surface antigen (HBsAg)/hepatitis B e antigen (HBeAg) positive] in these areas may be at increased risk of contracting HBV. The prevalence of new HBV infections among travellers is likely influenced by several factors, including the travellers’ immunity, the duration of their stay, the activities undertaken while abroad and the endemicity of the destination country.^8^ HIV infection is a major global public health concern that disproportionally affects developing countries.^9^ Travellers who engage in unprotected sexual intercourse in such countries are at increased risk of HIV acquisition.^10^ Importantly, evidence has demonstrated that the risk of HIV acquisition in travellers visiting countries with a low prevalence is higher in those visiting friends and relatives (VFRs) travellers.^11^ Though some countries have implemented restrictions on the mobility of persons living with HIV to prevent the spread of infection, the problem persists.^12^

The epidemiology of STIs is changing, driven by the emergence of infections that can be transmitted via sexual contact such as Zika virus infection^13^ and Mpox,^14^ the adoption of prevention measures such as pre-exposure prophylaxis (PrEP) of HIV^15^ and changes in patterns of sexual behaviour due to the rise of dating apps.^16^ This makes prevention and control strategies more challenging. There is also a concerning increase in multi-drug-resistant STIs, particularly mycoplasma genitalium^17^ and gonorrhoea.^18^ The resistance of gonorrhoea to almost all available antibiotics, including last-line therapeutic options,^19^ is becoming increasingly more widespread [i.e. resistance to azithromycin (18.6%) and decreased susceptibility to ceftriaxone (10.8%)],^20^ among travellers visiting Southeast Asia.^21^^,^^22^

International travel plays a major role in disseminating STIs and sexually transmitted BBVs worldwide (e.g. Mpox, Zika virus and HIV).^10^^,^^23^^,^^24^ Some individuals may see travel as an opportunity to escape from the social norms of daily life. This creates an environment that facilitates behaviour that one would not countenance at home.^25^ These changes in travellers’ sexual behaviour may increase the risk of acquisition of STIs and sexually transmitted BBVs.^26^^,^^27^ Furthermore, travelling can also accentuate pre-existing risk factors (e.g. history of multiple sexual partners and casual sex), thus increasing an individual’s susceptibility to acquiring STIs and sexually transmitted BBVs.^28^

The risk of STIs and sexually transmitted BBVs increases among travellers who engage in high risk behaviours, such as condomless sex, increasing numbers of partners, consuming excess alcohol and travelling for long periods.^29^^,^^30^ Evidence suggests that it is important to recognize that risk levels may be higher among some groups of travellers, such as men who have sex with men (MSM), adventure-seeking tourists, backpackers, VFRs, sex tourists and individuals travelling to regions with high STI and BBV rates.^31^^,^^32^ It is worth noting that VFRs constitute the largest group of travellers globally, and evidence has shown that VFRs face a higher risk of other travel-related infectious diseases.^33^

Identifying the specific risk profile within groups of travellers will be necessary to design targeted prevention measures to mitigate the risk of STIs and sexually transmitted BBVs. These interventions need to be tested and evidence based. For instance, this risk may be mitigated through appropriate, well-planned pre-travel counselling, such as recommending safer sexual practices, use of condoms, prescription of PrEP and post-exposure prophylaxis (PEP) for HIV and vaccination against HBV and hepatitis A virus (HAV).^34^^,^^35^ The rising incidence of STIs and BBVs among international travellers has been noted in some countries making this a priority area for investigation.^36^

From a preventive and control perspective, it is vital to first understand the extent to which international travel increases the risk of acquisition of STIs and BBVs and the factors contributing to their sexual acquisition. Therefore, we conducted a systematic review and meta-analysis to quantify the prevalence/proportionate morbidity of international travellers with STIs and sexually transmitted BBV*s* (i.e. *C. trachomatis*, gonorrhoea, syphilis, trichomoniasis, Zika virus, HAV, lymphogranuloma venereum [LGV], HIV, HBV and HCV) and the factors associated with the diseases in this group of individuals.

## Methods

### Protocol and registration

Reporting of the systematic review was done in accordance with the Preferred Reporting Items for Systematic Review and Meta-Analysis (PRISMA) recommendations (Supplementary material S1).^37^ The study protocol was prospectively registered in PROSPERO (registration number: CRD4202338885 7).

#### Variation in the protocol

Initially, the objective of the systematic review was to determine the acquisition rate of STIs among international travellers while overseas. Negative pre- and positive post-travel tests are needed to confirm the acquisition of STIs overseas. After extensive literature evaluation, studies did not follow this approach; instead, tests were conducted once, while overseas or upon return. Therefore, we modified the protocol (i.e. inclusion criteria) and the objective. We revised the protocol to focus on the prevalence/proportionate morbidity of international travellers diagnosed with STIs during or after travel, as we could not ascertain pre-departure STIs.

### Search strategy and databases

An experienced health sciences librarian (L.E.) developed a comprehensive search strategy to identify all relevant publications for the systematic review. Six electronic databases (i.e. PubMed, Scopus, Web of Science, CINAHL, Embase and Cochrane Library) were searched. The search strategy was designed to include studies published from inception of the databases to 14 November 2022. We used the following Medical Subject Headings (MeSH) terms and free text phrases in the search: ‘travellers’, ‘international travellers’, ‘abroad travellers’, ‘sexually transmitted disease’, ‘sexually transmitted infections’, ‘STIs’ and ‘STD’. Boolean operators were applied to combine the search terms. The specific search strategies for each database are included in Supplementary Material S2.

### Eligibility criteria and study selection

All records identified in the search strategy were imported into Endnote version 20 (Clarivate Analytics, Philadelphia, PA, USA),^38^ and duplicate records were removed. We then imported papers into Rayyan (Rayyan Systems Inc., Cambridge, MA 02142, USA; http://rayyan.qcri.org),^39^ for the screening process. Two reviewers (W.S. and B.M.M.) independently conducted the screening of articles in two stages, first by title and abstract and then by full text to determine its eligibility against predefined inclusion/exclusion criteria. The reasons for excluding full-text articles were recorded. Any disagreements between the reviewers at each stage of the selection process was resolved through discussion until consensus was reached. The reviewers presented substantial agreement (Cohen’s kappa = 0.8) throughout the screening process.

The inclusion criteria for the systematic review were:

- Analytical observational study design (i.e. cohort, case–control, cross-sectional).
- Studies that enrolled adult (>18 years of age) international travellers regardless of the reason for travel (e.g. business, tourism, study, adventure, VFRs) or mode of transportation.
- Studies that enrolled travellers who underwent screening for STIs and BBVs acquired/transmitted via sexual transmission, such as HIV, HBV, HCV, *C. trachomatis*, syphilis, gonorrhoea, Zika virus, Mpox, HAV, LGV and trichomoniasis, either at their destination or upon returning to their country of origin.
- Studies reported the prevalence/proportionate morbidity of international travellers with asymptomatic or symptomatic STIs and BBVs and the factors associated with sexual transmission of STIs and BBVs within this population. We estimated the proportionate morbidity of travellers with STIs for each category of travellers, utilizing studies that reported the number of STIs among ‘ill-travellers’ irrespective of cause of illness. Subsequently, we calculated the proportionate morbidity by dividing the reported cases of STIs by the total number of ill travellers for each included study. Prevalence was determined by dividing the number of reported STI cases by the total number of healthy or asymptomatic travellers in the study.

In the current review, we have included STIs and BBVs that can be transmitted/acquired via sexual activity, including emerging diseases (e.g. Zika virus and Mpox). We will refer to STIs and BBVs that can be transmitted/acquired via sexual activity as STIs from this point forward. However, it is important to note that some STIs have alternative modes of transmission beyond sexual contact (e.g. syphilis can transmit vertical from mother to child at any time during pregnancy, Zika virus is transmitted mostly through the bite of a mosquito and HBV can also be transmitted vertically^40^ and through infected blood or body fluid). To ascertain the sexual transmission of HBV, we considered the local epidemiological context of the birth countries of the individuals. Evidence suggests that the main transmission route is perinatal in regions where HBV is endemic, with a prevalence of over 2%.^41^ However, in non-endemic countries, sexual transmission of HBV has become common. Therefore, to confirm sexual transmission as the likely route, in addition to considering the local epidemiological context, we included studies that reported any high-risk sexual behaviours (i.e. sexual contact with an HBV-infected individual, MSM, having multiple sexual partners and prior or current STIs), excluding behaviours associated with injecting drug use.^6^

Similarly, there is evidence that HCV can be transmitted through sexual contact from individuals who are chronically infected to their heterosexual partners; however, this transmission mode is relatively infrequent, occurring at a rate of just one case per 190 000 sexual contacts.^42^ Furthermore, multiple studies have reported the sexual transmission of HCV among MSM living with and without HIV.^43^ Based on the available evidence, we initially intended to include HCV as a form of STI in studies involving MSM without a history of injecting drug use. However, as we could not confirm sexual transmission in the referenced articles, these were excluded.

Our review included studies that reported syphilis diagnoses based on clinical evaluation or serological tests (in the absence of clinical manifestations). By considering that sexual contact is the primary mode of syphilis acquisition/transmission and recognizing the epidemiological relevance of asymptomatic cases (i.e. asymptomatic patients are primarily treated to mitigate the risk of long-term complications of syphilis, rather than to decrease the risk of transmission for latent and late syphilis),^44^ the serologic criteria for these studies included the presence of non-treponemal test positivity [e.g. rapid plasma reagin (RPR) or the Venereal Disease Research Laboratory (VDRL) test], in combination with at least one reactive specific treponemal test [e.g. *Treponema pallidum* particle agglutination/haemagglutination (TPPA/TPHA)].^45^ Additionally, it is worth noting that multiple sources, including the European guideline on the management of syphilis, emphasizes that none of the serological tests differentiate between venereal syphilis and other non-venereal treponematoses.^46^ Non-venereal treponematosis, reported in tropical and subtropical regions (e.g. Africa and South America),^47^ makes it difficult for VFRs to determine where the infection was acquired due to limitations in diagnostic tests. The acquisition the infection during childhood makes it challenging to ascertain timing of syphilis infection through serologic tests. Therefore, in the case of VFRs, we have only considered studies that diagnosed syphilis in suggestive STI-related symptomatic travellers (i.e. only included those with symptoms of primary infection) and confirmed by laboratory diagnosis.

We imposed no restrictions on the language or year of publication. Studies were excluded if they only reported results on congenital STIs, domestic travellers, individuals who had sex with international travellers, immigrants or travellers residing in a foreign country for more than 1 year. Conference abstracts or proceedings, non-peer-reviewed or grey literature, retracted articles, studies on non-human hosts, case series, case reports, letters, commentaries and editorials were also excluded.

### Classification of travellers

Studies have shown that various travellers exhibit different risk levels for multiple health problems. Therefore, to determine the prevalence of STIs by type of travellers, we classify them into homogeneous groups by considering their characteristics and purpose of travel. For this review, we classified types of travellers into eight categories: (i) tourists, (ii) business travellers, (iii) students, (iv) VFRs, (v) backpackers, (vi) expatriates, (vii) international truck drivers and (viii) MSMs.

### Data extraction and quality assessment

The same two reviewers (W.S. and B.M.M.) independently extracted all relevant data from the included studies into a spreadsheet in Microsoft Excel (Microsoft, Redmond, WA, USA). The first reviewer (W.S.) conducted the data abstraction, while the second reviewer (B.M.M.) double-checked the extracted data.

The following characteristics from the studies were extracted: first/corresponding author, year of publication, country of origin of travellers, recruitment setting, study design, study period, number of participants, characteristics of the study population, symptomatic or asymptomatic travellers, mean/median duration of travel, country of destination, type of sample/diagnostic tests utilized, time frame for sample collection after travel and number positive cases or prevalence of travellers with STIs. Furthermore, information on the factors associated with STIs included socio-demographics (e.g. age, sex, education level, marital status, sexual orientation, occupation), trip details (e.g. the reason for travel, duration, origin and destination), preventive measures (e.g. attended pre-travel consultation) and sexual behaviour (e.g. number of sexual partners during travel, unprotected intercourse).

Quality assessment was conducted by the same two reviewers using a modified version of the assessment tool developed by Hoy *et al.*^48^ This tool comprises 10 items to assess the external (i.e. the representativeness of sample, sampling frame, selection of the sample and the response rate) and internal (i.e. the data collection methods, the case definitions, ensuring validity and reliability of tools, mode of data collection from the study subjects, appropriate prevalence period and correct numerators and denominators) validity of the studies. Each of the 10 items were scored as present or absent of bias for each study. We employed a continuous assessment approach across ten items in the risk of bias evaluation. After summarizing the overall evaluation using a bar chart, articles with a high score/percentage (i.e. ≥75%) were labelled ‘good’. It is important to note that ‘good’ is not an independent or absolute score; instead, it is a descriptive term used to emphasize comparisons within the items of risk of bias.

### Data synthesis and statistical analysis

The primary outcome of the current study was the prevalence/proportionate morbidity of international travellers with STIs, while the secondary outcome was the risk factors associated with STIs. We stratified the prevalence/proportionate morbidity estimates of each STIs by type of travellers (i.e. tourists, business travellers, students, VFRs, backpackers, expatriates, international truck drivers and MSMs). Quantitative data pooling (i.e. prevalence/proportionate morbidity meta-analysis) using the inverse variance heterogeneity model^49^ and the double arcsine transformations to stabilize the variance^50^ was conducted after grouping the data by the type of travellers. Subsequently, we employed subgroup analysis based on their clinical presentation (i.e. symptomatic vs asymptomatic) and types of STIs. Moreover, a narrative data synthesis of the included studies was carried out to summarize and give contextual information on the risk factors that contributed to STIs in travellers.

## Results

### Selection of studies

**Figure 1:**
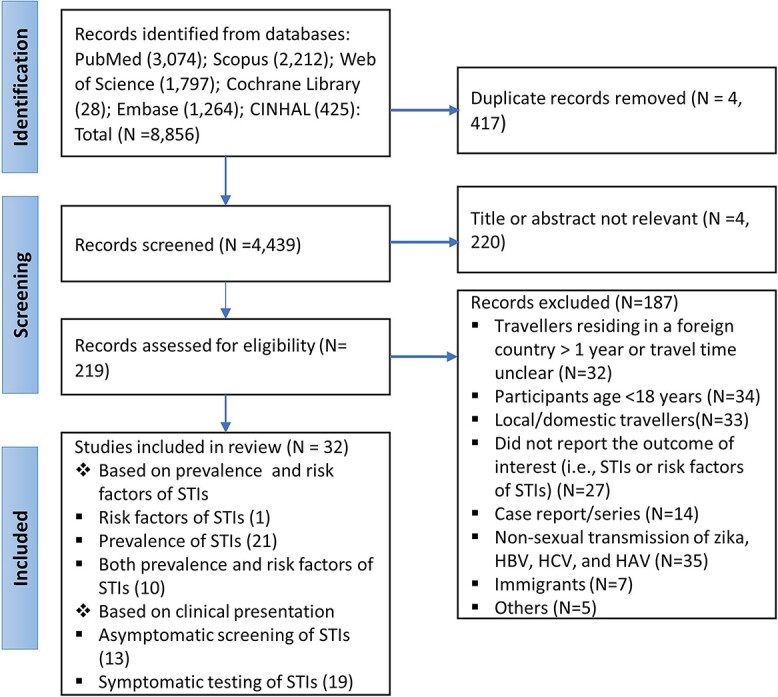
PRISMA flow chart to show the included studie

A total of 8856 records were retrieved [PubMed (*n =* 3074), Scopus (*n =* 2212), Web of Science (*n =* 1797), Cochrane Library (*n =* 28), Embase (*n =* 1264) and CINAHL (*n =* 425)]; of these, 4439 records remained after duplicates were removed. After the title and abstract screening, 219 publications underwent full-text screening. Thirty-two studies met the inclusion criteria and were included in the systematic review (Figure 1).

### Study characteristics

Overall, 32 studies with a total sample size of 387 731 international travellers were included. The number of travellers per study ranged from 152^51^ to 102 622.^52^ Nineteen (*n =* 350 389) studies evaluated the proportionate morbidity of STIs among symptomatic travellers, while 13 (*n =* 37 342) studies assessed the prevalence of STIs in asymptomatic travellers. Apart from one study, conducted as a case–control study,^53^ all used a cross-sectional study design. All studies reported participant country of origin; 19 (59.4%) studies included travellers from multiple countries,^52–70^ four (12.5%) from the UK,^54^^,^^58^^,^^69^^,^^71^ three (9.4%) from the USA,^72–74^ two (6.3%) from Sweden,^75^^,^^76^ and one (3.1%) each from Finland,^77^ Belgium,^51^ Malta,^78^ Canada,^79^ Switzerland,^80^ North Korea^81^ and France.^82^ The median age of the participants ranged from 21^73^ to 45^59^ years. Destination of travel was reported in 30 studies, with Asia (*n =* 17, 53.1%)^52^^,^^53^^,^^55^^,^^59–61^^,^^63^^,^^65^^,^^67^^,^^69^^,^^71^^,^^75–77^^,^^80–82^ and Africa (*n =* 12, 37.5%)^51^^,^^52^^,^^54^^,^^55^^,^^60^^,^^63^^,^^69^^,^^71^^,^^73^^,^^76^^,^^80^^,^^82^ being the most common travel destinations. More than one-third (*n =* 11, 34.4%) of the studies included travellers to multiple destinations. Travel duration in the studies ranged from 6^60^ to 44 weeks.^72^ More than two-third (*n =* 22, 68.7%) of the studies did not report the time spent overseas by the travellers.

Thirteen (40.6%) studies screened for HIV*,*^52^^,^^54^^,^^55^^,^^59–65^^,^^70^^,^^73^^,^^82^ 10 (31.3%) for syphilis*,*^52^^,^^55^^,^^61^^,^^63^^,^^65^^,^^70^^,^^73^^,^^77^^,^^80^^,^^81^ six (18.7%) for HBV*,*^61^^,^^65^^,^^72^^,^^76^^,^^80^^,^^81^ seven (21.8%) for *C. trachomatis,*^56–58^^,^^66^^,^^71^^,^^73^^,^^75^ seven (21.8%) for gonorrhoea*,*^52^^,^^53^^,^^70^^,^^71^^,^^77^^,^^78^^,^^80^ four (12.5%) for Zika virus,^67^^,^^69^^,^^74^^,^^79^ two (6.3%) for LGV,^70^^,^^78^ one (3.1%) for HAV^68^ and eight (25.0%) did not specify the type of STIs.^52^^,^^54^^,^^60^^,^^63^^,^^70^^,^^71^^,^^77^^,^^82^ On the other hand, despite a comprehensive literature search, we did not find any published articles on MPox in travellers, except for a few case reports. In 14 (43.7%) studies, the diagnosis of STIs was performed using blood samples,^51^^,^^59^^,^^61^^,^^62^^,^^65^^,^^67^^,^^68^^,^^71–73^^,^^75^^,^^76^^,^^79^^,^^80^ in three (9.4%) studies, urine samples were used,^54^^,^^57^^,^^66^ in one (3.1%) study, rapid oral HIV tests were utilized,^64^ three (9.4%) studies used both urine and blood samples,^69^^,^^74^^,^^77^ one (3.1%) urine and rectal sample^78^ and in one (3.1%) study, men provided a urine sample and women provided a self-collected vaginal swab.^56^ The diagnostic procedure utilized in the remaining four (12.5%) studies was not reported.^53^^,^^55^^,^^60^^,^^70^ Thirteen (40.6%) studies screened asymptomatic travellers,^51^^,^^53^^,^^56^^,^^57^^,^^59^^,^^62^^,^^64–66^^,^^72^^,^^75^^,^^76^^,^^81^ while 19 (59.4%) studies included symptomatic travellers.^52^^,^^54^^,^^55^^,^^58^^,^^60^^,^^61^^,^^63^^,^^67–71^^,^^73^^,^^74^^,^^77–80^^,^^82^ Nineteen (59.4%) studies tested for STIs after participants had returned from overseas,^51–55^^,^^60^^,^^63^^,^^67–72^^,^^74^^,^^75^^,^^77–80^ while nine (28.1%) studies reported results at the country of destination (i.e. while overseas).^56–59^^,^^61^^,^^62^^,^^64^^,^^65^^,^^81^ The characteristics of the included study are presented in Table 1.

**Table 1:** Summary of the characteristics of included studies

Author, year	Country of origin	Recruitment setting [study period]	Median age (in years)	Study population and sample size	Classified in our study as	Travel destination [median travel duration]	Type of sample and testing upon return/overseas [type of test] [STIs or sexually transmitted BBVs tested]
Asymptomatic clinical presentation							
Alzahrani AJ *et al.*, 2009^62^	India, Bangladesh, Pakistan, Indonesia, Sri Lanka, Nepal, Thailand and Sudan	Pre-employment testing in Saudi Arabia [NR]	30^*^	875 expatriate workers	Expatriates	Saudi Arabia [NR]	Blood, tested upon arrival [WB] [HIV]
Beauté J *et al.*, 2017^53^	Denmark, Finland, Norway and Sweden	Epidemiologic surveillance data from four Nordic countries [2008–13]	33	12 645 travellers	Tourists	Europe, Asia and North America [NR]	[NR] Surveillance data done upon return [NR], [Gonorrhoea]
Bonneux L *et al.*, 1988^51^	Belgium	Outpatient clinic of Tropical Medicine in Antwerp [1985–87]	41*	5965 return expatriates	Expatriates	Central Africa [6 months]	Blood, test done upon return [antibody test, confirmed by indirect immunofluorescence and immunoblotting] [HBV, HIV]
Botros B *et al*., 2009^59^	Enrolled from 21 countries, majority (Turkey, Russian, Georgia and Ukraine)	Two clinics at major truck terminals in Baku [2004–05]	45	3763 international truck drivers	International truck driver	Azerbaijan [NR]	Blood, test done overseas [immunochromatographic rapid test for the qualitative detection of antibodies to HIV] [HIV]
Chimungu B *et al.*, 2020^65^	Europe, Asia, North America, Africa, Oceania and South America	International Travel Health Care Centre [2010–17]	33*	40 935 foreigners arriving in China within 6 months	Business travellers/students	China [6 months]	Blood, test done overseas within 6 months of arrival, [HbsAg, anti-HIV, anti HCV, TPPA] [HBV, HIV and syphilis]
Davies S *et al.*, 2011^56^	Europe (UK, Scandinavia, Ireland, Germany and France), America and Asia	Hostels in four areas popular with young international travellers [2009]	23	432 international backpackers	Backpackers	Australia [4 months]	Urine (men) within a median of three months and vaginal swab (women) within a median of two months, test done overseas [DNA BD Viper] [chlamydia]
Decraene V *et al.*, 2018^75^	Sweden	National surveillance system [2000–13]	23	[NR] travellers who had one or more overnight travels outside Sweden	Tourists	Europe, Asia and North America [NR]	Blood, tested upon return [NAAT] [*C*. *trachomatis*]
Fischer JA *et al.*, 2015^57^	UK and Ireland	Community health venue located near many backpackers [2010]	23	160 international backpackers	Backpackers	Australia [90 days]	Urine, tested within a median of 90 days in overseas [PCR] [*C. trachomatis*]
Han P *et al.*, 2019^81^	North Korea	China through Dandong port [2015–17]	NR	18 494 travellers	Business travellers/students	China [NR]	Blood, tested upon arrival in overseas [PCR/TRUST, TPPA] [HBV and syphilis]
Kramer MA *et al.*, 2008^64^	Surinamese and Antillean	Social venues in two large cities [2003–05]	32	1092 VFRs in the Netherlands	VFRs	Netherland [NR]	Saliva, test done overseas [Ab] [HIV]
Struve J *et al.*, 1995^76^	Sweden	Clinic [1991]	44	563 expatriates	Expatriates	Africa, Asia, Europe, America and Australia [NR]	Blood, tested upon return [HBsAg] [HBV]
Trevis T *et al.*, 2018^66^	Europe, Asia, Oceania, North and south America	Hostel-style accommodation [2016]	23	271 international backpackers	Backpackers	Australia [NR]	Urine, test done in overseas [PCR] [gonorrhoea, C*. trachomatis*]
Truong H-HM *et al.*, 2018^72^	USA	San Francisco Bay Area [2009–11]	NR	478 MSM with travel history in the past 12 months	MSMs	NR	Blood, tested within 12 months of return [HBcAb, rapid Ab serologic] [HBV, HIV]
Symptomatic clinical presentation							
Angelo KM *et al.*, 2018^73^	USA	GeoSentinel sites in the USA [2007–17]	21	432 returned students	Students	Sub-Saharan Africa, South and Central America [40 days]	Angelo KM *et al.*, 2018^73^
Angelo KM *et al.*, 2020^67^	Dominican Republic, Americas and Caribbean	GeoSentinel sites [2012–19]	36	525 international travellers	Tourists	North America, Central America, South America Caribbean and Southeast Asia [17 days]	Angelo KM *et al.*, 2020^67^
Ansart S *et al*., 2005^82^	France	Tropical diseases unit in France [2002–03]	38	622 returned travellers	Tourists/business travellers/expatriates	Africa, Asia, Oceania, South and Central America [37 days]	Ansart S *et al*., 2005^82^
Boggild AK *et al.*, 2017^79^	Canada	CanTravNet sites [2015–16]	36	1 128 returned travellers	Tourists	Americas (Caribbean, north, south and Central America) [15 days]	Boggild AK *et al*., 2017^79^
Donachie A *et al.*, 2018^78^	Malta	Genitourinary clinic [2018]	NR	48 returned MSM travellers	MSMs	NR	Rectal, urine, tested upon return [NAATs, serologic] [LGV, HIV, gonorrhoea]
Fang LQ *et al.*, 2018^61^	Africa, Western Pacific, South-East Asia, Europe and America	All international entry–exit ports in each of the 22 provinces [2014–16]	30	22 797 travellers in overseas	Tourist/business travellers/students/VFRs	China [NR]	Blood, test conducted overseas upon arrival [ELISA/WB/RIBA, TPPA] [HIV, syphilis]
Field V *et al.*, 2010^63^	Europe	EuroTravNet core site clinics [2008]	36	6 302 returned travellers	Tourists/business travellers/students/VFRs	Sub Saharan Africa, Southeast Asia, Middle East, South and Central America [29 days]	Blood, tested upon return [NR] [HIV, syphilis and unspecified STI]
Hawkes S *et al.*, 1995^71^	UK	Genitourinary medicine clinic [1993]	30*	315 returned travellers attending sexual health clinic	Tourists	Asia and Sub-Saharan Africa [NR]	Blood, within 3 months of return [serology] [gonorrhoea, *C*. *trachomatis* and unspecified STIs]
Hawkes SJ *et al.*, 1994^54^	UK	Hospital for tropical disease [1991–1992]	30*	731 travellers visiting hospital	Tourists	North America, Western Europe, Australia & Sub Sharan Africa [3 months]	Urine, within 6 weeks of return [WB, serology] [HIV and unspecified STIs]
Matteelli A *et al.*, 2013^52^	Africa, Asia, South America, Caribbean and eastern Europe	GeoSentinel clinics worldwide [1996–2010]	40*	112 180 retuned travellers	Tourists/business travellers/students/VFRs	Southeast Asia, Sub-Saharan Africa, South America and Western Europe [1 month]	[NR], microbiological and clinical evaluation upon return [NR] [HIV, syphilis, gonorrhoea and unspecified STIs]
McNulty A *et al.*, 2010^58^	UK	Sydney sexual health centre [1998–2006]	25	5702 international backpackers	Backpackers	Australia [NR]	[NR], test done overseas [NR] [*C*. *trachomatis*]
Ndumbi P *et al.*, 2018^68^	Europe	Local public health departments [2016–17]	33	195 MSM returned travellers	MSMs	Spain, Germany, Belgium and Portugal [NR]	Blood, tested upon return [anti-HAV IgM, PCR] [HAV]
Petridou C *et al*., 2019^69^	UK	RIPL laboratory [2016–17]	NR	6333 returned travellers	Tourists	Caribbean, South America, Southeast Asia and Africa [NR]	Urine & blood, tested upon return [PCR, ZIKV RNA, ZIKV IgM/G] [Zika virus]
Porse CC *et al.*, 2018^74^	USA	Local health departments in California [2015–17]	NR	588 returned travellers	Tourists	México, Central America and Caribbean [NR]	Urine, semen, blood, tested upon return [PCR, ZIKV IgM/G] [Zika virus]
Schlagenhauf P *et al.*, 2015^60^	Europe	Euro TravNet clinics [2008–12]	35	12 870 returned travellers	Tourists	Sub-Saharan Africa, Asia and South America [44.5 days]	Blood, test done upon return [NR] [HIV and unspecified STI]
Steffen R, *et al*., 1987^80^	Switzerland	Survey upon return from developing countries [1981–84]	40	7886 returned travellers	Tourists	Asia and Africa [3 weeks]	Blood, test done upon return [serology] [HBV, gonorrhoea, syphilis]
Wilson ME, 2014^70^	Europe, Asia, Oceania, North and south America	GeoSentinel Clinic sites [1997–2013]	NR	1586 returned travellers	Tourists	Brazil [NR]	[NR], microbiological and clinical evaluation upon return [NR] [HIV, syphilis, LGV, gonorrhoea and unspecified STIs]
Wu Y *et al.*, 2020^55^	China and others (Africa, Oceania, Asian and Latin American)	International entry–exit ports [2014–18]	34	58 677 returned travellers	Tourists/business travellers/students/VFRs	Asia, Africa, Europe, North America and Oceania [NR]	Blood, tested upon return [ELISA/WB/RIBA/TRUST/TPPA/PCR] [HIV, syphilis, gonorrhoea, *C*. *trachomatis* and trichomoniasis]
Zöldi V *et al.*, 2018^77^	Finland	Travel data extracted from Finland database [1995–2015]	NR	73 233 travellers return from overseas	Tourists	Asia and Oceania [NR]	Blood and urine, tested upon return [antibody] [gonorrhoea and syphilis]

Abbreviations: Ab: antibody; Ag: antigen; ELISA: enzyme-linked immunosorbent assay; HB(c/s)Ag: hepatitis B core/surface antigen; HBV: hepatitis B virus; HIV: human immunodeficiency virus; NAAT: nucleic acid amplification tests; NIDR: National Infectious Diseases Register; NNIDRS: National Notifiable Infectious Disease Reporting System; NR: not reported; PCR: polymerase chain reaction; PRNT: plaque reduction neutralization test; RIBA: recombinant immunoblot assay; RPR: rapid plasma reagin; TPPA: *T. pallidum* particle assay; TPHA: *T. pallidum* haemagglutination assay; TRUST: tolulized red unheated serum tests; WB: western blot; USA: United States of America; *mean age of the study participants.

### Prevalence/proportionate morbidity of international travellers with STIs

Overall, 31 studies reported on the prevalence/proportionate morbidity of travellers with STIs.^51^^,^^52^^,^^54–77^^,^^79–82^ The prevalence of asymptomatic and symptomatic travellers with STIs compared to the type of traveller is summarized in Table 2 and the Supplementary material S3.

**Table 2:** The prevalence/proportionate morbidity of symptomatic and asymptomatic travellers with STIs by the type of travellers

STIs	Type of traveller							
	Tourists	Students	Business travellers	VFRs	Backpackers	Expatriates	International truck drivers	MSMs
	Prevalence(95% CI) [number of studies]	Prevalence(95% CI) [number of studies]	Prevalence(95% CI) [number of studies]	Prevalence(95% CI) [number of studies]	Prevalence(95% CI) [number of studies]	Prevalence(95% CI) [number of studies]	Prevalence(95% CI) [number of studies]	Prevalence(95% CI) [number of studies
Symptomatic travellers [proportionate morbidity]								
HIV	0.28(0.09–0.56)^52^^,^^54^^,^^60^^,^^61^^,^^63^^,^^70^^,^^82^ [*n =* 7]	0.93(0.00–4.31)^63^^,^^73^ [*n =* 2]	0.14(0.02–0.77)^63^ [*n =* 1]	0.72(0.33–1.57)^63^ [*n =* 1]	—	—	—	2.50(0.44–12.88)^78^ [*n =* 1]
HBV	1.15(0.00–4.81)^61^^,^^80^ [*n =* 2]	—	—	—	—	—	—	—
HAV	—	—	—	—	—	—	—	20.0(14.99–26.17)^68^ [*n =* 1]
Syphilis	0.44(0.04–1.15)^52^^,^^61^^,^^70^^,^^71^^,^^77^^,^^80^ [*n =* 6]	0.23(0.04–1.30)^73^ [*n =* 1]	—	1.67(1.01–2.81)^63^ [*n =* 1]	0.02(0.00–0.01)^58^ [*n =* 1]	—	—	—
*Chlamydia trachomatis*	1.59(0.68–3.66)^71^ [*n =* 1]	0.23(0.04–1.30)^73^ [*n =* 1]	—	—	6.58(5.96–7.25)^58^ [*n =* 1]	—	—	—
Gonorrhoea	0.95(0.00–3.73)^52^^,^^71^^,^^77^^,^^80^ [*n =* 4]	—	—	—	0.72(0.53–0.97)^58^ [*n =* 1]	—	—	4.17((1.15–13.98)^78^ [*n =* 1]
Trichomoniasis	0.02(0.00–1.15)^52^^,^^71^ [*n =* 2]	—	—	—	0.07(0.03–0.18)^58^ [*n =* 1]	—	—	—
LGV	0.09(0.00–0.25)^70^^,^^71^ [*n =* 2]	—	—	—	—	—	—	4.17((1.15–13.98)^78^ [*n =* 1]
Zika virus	0.08(0.0–00.58)^67^^,^^69^^,^^74^^,^^79^ [*n =* 4]	—	—	—	—	—	—	—
Unspecified	2.40(0.00–6.60)^52^^,^^54^^,^^55^^,^^61^^,^^70^^,^^82^ [*n =* 6]	1.02(0.00–5.63)^52^^,^^55^^,^^61^ [*n =* 4]	1.73(0.00–8.94)^52^^,^^55^^,^^61^^,^^82^ [*n =* 4]	1.62(0.00–7.10)^52^^,^^55^^,^^61^ [*n =* 3]	—	4.76(1.32–15.79)^82^ [*n =* 1]	—	—
Asymptomatic travellers [prevalence]								
HIV	—	0.02(0.01–0.07)^65^ [*n =* 1]	0.01(0.00–0.06)^65^ [*n =* 1]	0.37(0.14–0.94)^64^ [*n =* 1]	—	0.75(0.0–2.01)^51^^,^^62^ [*n =* 2]	1.54(1.14–1.93)^59^ [*n =* 1]	25.94(22.21–30.05)^72^ [*n =* 1]
HBV	—	1.76(0.26–4.28)^65^^,^^81^ [*n =* 2]	1.56(0.00–5.03)^65^^,^^81^ [*n =* 2]	—	—	0.31(0.14–0.55)^51^^,^^76^ [*n =* 2]	—	24.90(21.23–28.96)^72^ [*n =* 1]
HAV	—	—	—	—	—	—	—	—
Syphilis	—	0.43(0.00–1.22)^65^^,^^81^ [*n =* 2]	0.74(0.00–2.24)^65^^,^^81^ [*n =* 2]	—	—	0.11(0.05–0.26)^51^ [*n =* 1]	—	—
*C. trachomatis*	0.02(0.00–0.03)^75^ [*n =* 1]	—	—	—	3.92(2.72–5.32)^56^^,^^57^^,^^66^ [*n =* 3]	—	—	—
Gonorrhoea	—	—	—	—	0.00(0.00–0.05)^57^^,^^66^ [*n =* 2]	—	—	—
Trichomoniasis	—	—	—	—	—	—	—	—
LGV	—	—	—	—	—	—	—	—
Zika virus	—	—	—	—	—	—	—	—
Unspecified	—	—	—		—	—	—	—

Abbreviations: CI: confidence interval; HAV: hepatitis A virus; HBV: hepatitis B virus; HCV: hepatitis c virus; HIV: human immunodeficiency virus; LGV: lymphogranuloma venereum; MSM: men who have sex with men; STIs: sexually transmitted infections; VFRs: visiting friends and relatives

### Symptomatic international travellers

#### Tourists

Sixteen (50.0%) studies reported on tourists travellers (*n =* 299 915),^52^^,^^54^^,^^55^^,^^60^^,^^61^^,^^63^^,^^67^^,^^69–71^^,^^74^^,^^75^^,^^77^^,^^79^^,^^80^^,^^82^and it was noted that the proportionate morbidity of symptomatic tourist travellers with STIs was higher (e.g. HIV [0.28%; 95% CI: 0.09–0.56%], HBV [1.15%;95% CI: 0.00–4.81%], *C. trachomatis* [1.59%; 95% CI: 0.68–3.66%], syphilis [0.44%; 95% CI: 0.04–1.15%], gonorrhoea [0.95%; 95% CI: 0.00–3.73%] and Zika virus [0.08%; 95% CI: 0.00–0.58%]) than in symptomatic student travellers.

#### Students

Five (15.6%) studies reported on symptomatic students (*n =* 3568),^52^^,^^55^^,^^61^^,^^63^^,^^73^ and the proportionate morbidity of symptomatic students with STIs was lower (i.e. syphilis [0.23%; 95% CI: 0.04–1.30%], *C. trachomatis* [0.23%; 95% CI: 0.04–1.30%] and unspecified STIs [1.02%; 95% CI: 0.00–5.63%]), compared to other type of symptomatic travellers, except for HIV (0.93%; 95% CI: 0.00–4.31%).

#### Business travellers

Five (15.6%) studies reported on business travellers (*n =* 31 053),^52^^,^^55^^,^^61^^,^^63^^,^^82^ and the proportionate morbidity of symptomatic business travellers with unspecified STIs was higher 1.73% (95% CI: 0.00–8.94%) than in symptomatic student travellers.

#### VFRs

Four (12.5%) studies reported on VFRs travellers (*n =* 9388),^52^^,^^55^^,^^61^^,^^63^ and the proportionate morbidity of symptomatic VFRs with STIs was higher (e.g. syphilis [1.67%; 95% CI: 1.03–2.81%] and unspecified STIs [1.62%; 95% CI: 0.00–7.10%]) than in symptomatic student travellers.

#### Backpackers

One (3.1%) study reported on backpackers (*n =* 5702),^58^ and the proportionate morbidity of symptomatic backpackers with *C. trachomatis* (6.58%; 95% CI: 5.96–7.25%) was higher than in the other groups of symptomatic travellers.

#### Expatriates

One (3.1%) study reported on expatriate travellers (*n =* 42),^82^ and the proportionate morbidity of symptomatic expatriates with unspecified STIs (4.76%; 95% CI: 1.32–15.79%) was higher than in the other groups of travellers.

#### MSMs

Two (6.2%) studies reported on MSM travellers (*n =* 721),^68^^,^^78^ and the proportionate morbidity of symptomatic MSM travellers with STIs was higher (i.e. HIV [2.50%; 95% CI: 0.44–12.88%], gonorrhoea [4.17%; 95% CI: 1.1.5–13.98%], LGV [4.17%; 95% CI: 1.1.5–13.98%] and HAV [20.0%; 95% CI: 14.99–26.17%]) than in the other groups of symptomatic travellers. Notably, these studies exclusively focused on MSM tourists.

### Asymptomatic international travellers

#### Tourists

One (3.1%) study reported on tourist travellers,^75^ and the prevalence of asymptomatic tourist travellers with STIs was lower (i.e. *C. trachomatis* [0.02%; 95% CI: 0.00–0.03%] than in other types of asymptomatic travellers.

#### Students

Two (6.2%) studies reported on student travellers (*n =* 12 575),^65^^,^^81^and the prevalence of asymptomatic student travellers with STIs was higher (e.g. HBV [1.76%; 95% CI: 0.26–4.28%], syphilis [0.43%; 95% CI: 0.00–1.22%]) than in asymptomatic tourist travellers. Notably, one study^81^ reported zero prevalence of syphilis among asymptomatic students, which may contribute to a higher combined effect size estimate.

#### Business travellers

Two (6.2%) studies reported on business travellers (*n =* 11 210),^65^^,^^81^ and the prevalence of asymptomatic business travellers with STIs was higher for syphilis (0.74%; 95% CI: 0.00–2.24%) than in the other groups of asymptomatic travellers. Additionally, the prevalence of asymptomatic business travellers with HBV was 1.56% (95% CI: 0.00–5.03%).

#### VFRs

One (3.1%) study reported on VFRs travellers (*n =* 1092),^64^and the prevalence of asymptomatic VFRs with STIs was higher for HIV (0.37%; 95% CI: 0.14–0.94%) than in the most groups of asymptomatic travellers.

#### Backpackers

Three (9.4%) studies reported on backpackers’ travellers (*n =* 863),^56^^,^^57^^,^^66^ and the prevalence of asymptomatic backpackers with *C. trachomatis* was higher (3.92%; 95% CI: 2.72–5.32%) than in the other groups of asymptomatic travellers.

#### Expatriates

Three (9.4%) studies reported on expatriates (*n =* 7361),^51^^,^^62^^,^^76^ and the prevalence of asymptomatic expatriates with HIV was higher (0.75%; 95% CI: 0.00–2.01%) than in the most groups of asymptomatic travellers, except MSM travellers. Additionally, the prevalence of asymptomatic expatriate travellers with syphilis and HBV was found to be 0.11% (95% CI: 0.05–0.26%) and 0.31% (95% CI: 0.14–0.55%), respectively.

#### International truck drivers

One (3.1%) study reported on truck drivers (*n =* 3763),^59^ and the prevalence of asymptomatic truck drivers with HIV was 1.5% (95% CI: 1.14–1.93%).

#### MSMs

One (3.1%) study reported on MSM travellers (*n =* 478),^72^ and the prevalence of asymptomatic MSM travellers with STIs was higher (e.g. HBV [25.0%; 95% CI: 21.23–28.96%], HIV [26.0%; 95% CI: 22.21–30.05%]) than in the other groups of asymptomatic travellers.

### Factors associated with STIs among international travellers

Eleven (34.4%) studies reported on the factors associated with STIs and sexually transmitted BBVs.^51–54^^,^^56^^,^^57^^,^^59^^,^^65^^,^^66^^,^^71^^,^^72^ The specific risk factors associated with sexual transmission of STI and BBV and by type of traveller are presented in Table 3.

**Table 3:** Risk factors for STIs among international travellers

STIs	Type of traveller						
	Tourists	Business travellers/students	VFRs	Backpackers	Expatriates	International truck drivers	MSMs
HIV	Associated: Prior STIs^54^ Not associated: visited sexual clinic,^54^ Contradicting evidence: Origin of the region (Africa^54^)		Associated: male,^64^ CSW^64^ and frequent visit of country of origin^64^ Not associated: married/cohabiting^64^	—	Associated: multiple sexual partners,^51^ sexual with CSW,^51^ sexual with local women,^51^ low education level^51^ Not associated: oro-genital or anorectal sex^51^	Associated: residence Russia,^59^ IDU,^59^ prior STIs,^59^ Unmarried,^59^ MSM^59^ Not associated: age,^59^ sex with CSW,^59^ circumcision^59^	—
HBV	—	Associated: old age (>50 years),^65^ lower education level,^65^ occupation (businessmen and students): (have lower risk^65^)	—	—	Not associated: having sexual contact with locals^76^	—	Associated: age,^72^ not vaccinated,^72^ HIV^72^ Not associated: travel to HBV endemic country,^72^ race/ethnicity^72^
Syphilis	—	Associated: region of the origin,^65^ old age(>50 years),^65^ uneducated,^65^ businessmen and students^65^ Not associated: sex^65^	—	—	—	—	—
Gonorrhoea	Associated: Country of residence (Norway, Sweden, and Finland),^53^ male sex,^53^ old age (>65 years),^53^ heterosexual^53^	—	—	—	—	—	—
*C. trachomatis*	—	—	—	Associated: stay overseas for longer,^56^ staying at a beachside^56^ Not associated: alcohol use,^56^ travel with partner,^56^ sexual orientation,^56^ prior chlamydia^56^^,^^57^ and unprotected sex^56^ Contradicting evidence: multiple sexual partners^57^^,^^66^	—	—	—
Unspecified	Associated: Short duration of travel,^52^ visited sexual clinic,^71^ not having pretravel advice,^52^ region of travel,^52^ VFR^52^ Not associated: Solo travel,^71^ Homosexual^71^ Contradicting evidence: Sex (no association,^71^ male^52^)	—	—	—	—	—	—

Abbreviations: CSW: commercial sex worker; HBV: hepatitis B virus; HCV: hepatitis c virus; HIV: human immunodeficiency virus; IDU: injected drug user; MSM: men who have sex with men; STIs: sexually transmitted infections; VFRs: visiting friends or relatives.

#### Tourists

One study reported on HIV,^54^ and prior STIs (odds ratio [OR]: 6.27; 95% CI: 4.95–7.59) were the only significant independent risk factors.^54^ Another study^53^ reported on gonorrhoea, and the risk factors were heterosexual men (OR: 4.08; 95% CI: 3.66–4.56) and travellers originating from Norway (OR: 1.48; 95% CI: 1.26–1.73) and Finland (OR: 1.37; 95% CI: 1.15–1.62) compared with Sweden. Two studies^52^^,^^71^ reported on unspecified type of STIs, and the risk factors were duration of travel < 1 month (OR: 1.25; 95% CI: 1.01–1.56), VFRs (OR: 2.12; 95% CI: 1.62–2.78), not having received professional pre-travel health advice (OR: 1.50; 95% CI: 1.20–1.87), travel to Southeast Asia (OR: 4.34; 95% CI: 2.71–6.96), Sub-Saharan Africa (OR: 2.32; 95% CI: 1.45–3.73) and South America (OR: 3.07; 95% CI: 1.80–5.25).

#### Business travellers/students

One study reported on syphilis,^65^ and the risk factors were business-type travellers (OR: 3.02; 95% CI: 2.03–4.49), students (OR: 1.98; 95% CI: 1.29–3.06) and travellers who originated from Europe (OR: 7.34; 95% CI: 3.53–15.27), North (OR: 5.00; 95% CI: 2.34–10.68) and South America (OR: 19.30; 95% CI: 8.81–42.29) compared with Asia.

#### VFRs

One study reported on HIV,^64^ and the risk factors were men (OR: 2.33; 95% CI: 1.34–3.14), frequent number of visits to the country of origin in the past 5 years (OR: 2.1; 95% CI: 1.56–2.94) and having sexual contact with a commercial sex worker (CSW) (OR: 2.05; 95% CI: 1.46–2.88).

#### Backpackers

Three studies reported on *C. trachomatis*,^56^^,^^57^^,^^66^ and the risk factors were staying at a beachside hostel (OR: 3.19; 95% CI: 0.88–11.6)^56^ and travelling for more than 4 months (OR: 2.63; 95% CI: 0.83–8.40) (the study’s cut-off value to ensure the significance levels were *P* < 0.1).^56^ However, no association were found for alcohol use, unprotected sex, multiple sexual partners and travelling with a partner.

#### Expatriates

One study reported on HIV,^51^ and the risk factors were having prior STIs (OR: 8.30; 95% CI: 2.80–25.20), engaging with multiple sexual partners (OR: 7.10; 95% CI: 1.2–42.0), having sexual contact with a CSW (OR: 10.80; 95% CI: 1.60–71.90) and sexual contact with local women (OR: 14.7; 95% CI: 2.81–76.90).

#### International truck drivers

One study examined factors associated with HIV^59^ and found that Russian truck drivers (OR: 2.23; 95% CI: 1.14–4.37), MSM (OR: 49.77; 95% CI: 8.61–270.99), being single (OR: 2.79; 95% CI: 1.25–9.19), unprotected sex while travelling (OR: 5.0; 95% CI: 2.61–9.10) and having prior ‘STIs’ (OR: 4.66; 95% CI: 2.60–8.36) were associated with an increased odds of HIV infection.

#### MSMs

One study reported on HBV,^72^ and the risk factors were having concurrent HIV infection (OR: 2.43; 95% CI: 1.77–3.33), not being vaccinated (OR: 2.23; 95% CI: 1.61–3.41) and older age (OR: 1.49; 95% CI: 1.31–1.70). However, there was no statistical association between travelling to an HBV-endemic country (i.e. the national prevalence of HBV surface antigen in birth or visited country was ≥8%) and race/ethnicity.

### Quality assessment

Overall, several articles received good scores, including uniform data collection methods for all participants (*n =* 32, 100.0%), appropriate case definitions (*n =* 30, 93.7%), validation and reliability of study tools (*n =* 29, 90.6%), the length of shortest prevalence period (*n =* 29, 90.6%) and direct data collection from participants (*n =* 24, 75.0%). However, deficiencies were observed in components related to external validity, such as the representativeness of the population (*n =* 11, 34.4%) and the potential for non-response bias (*n =* 12, 37.5%). The details of the risk of bias assessment are reported in Supplementary materials S4 and S5*.*

## Discussion

This systematic review and meta-analysis revealed that the prevalence/proportionate morbidity of travellers with STIs varied across the different groups of travellers, with lower rates among business travellers and higher rates in backpackers and VFRs with significantly higher rates among MSM travellers. Similarly, our review found that the risk of STIs in travellers varies depends on factors such as the destination (e.g. regions like Southeast Asia showing a higher risk) and the impact of pre-travel consultation and vaccination against HBV, which contributes to decreasing the risk of both STIs and sexually transmitted BBVs. Our findings are in line with a previous review that suggests international travellers who had casual sexual encounters were associated with an increased risk of acquiring STIs.^30^ Additionally, our findings align with Australia’s fourth national STIs strategy framework, which has identified travellers as a priority population due to their increased risk of STI acquisition and the potential impact of onward transmission while travelling in a country.^83^ Moreover, the Public Health Agency of Canada recommends considering an individual’s travel history as an integral part of the risk assessment for STIs.^84^

The variation in STI prevalence/proportionate morbidity among different groups of travellers may be attributed to the specific characteristics of each traveller. For instance, MSM travellers were found to have a high prevalence of STIs (>25%). This is not an unexpected finding as MSM have been found to have higher rates of STI diagnosis than other population groups in general;^85^ however, it is of note that the available evidence included in this review is insufficient to draw a firm conclusion regarding the specific reasons for this result as the finding came from only one study.^72^ Further research is needed in this area. It was observed that MSM who received HBV vaccination before travelling had a lower risk of infection.^86^ This is not an unexpected finding. It is of note, however, that the included study only tested for hepatitis B core antibody, which does not differentiate between acute and chronic HBV infection. The inclusion of possible chronic carriers could lead to overestimating the risk of HBV acquisition. Similarly, our review identified a high prevalence/proportionate morbidity of backpackers with STIs, with the majority being young, sexually active males who outnumbered other travellers. These populations have been found to engage in sexual behaviours that place them at an increased risk of STI acquisition;^87^ however, despite this known risk, they don’t get checkups or if they get symptoms, they don’t get treatment while travelling due to language barriers and cultural differences.^88^ Traveller often cross multiple countries before reaching their intended destinations,^89^ which can facilitate wider spread of STIs. Financial constraints may discourage travellers from seeking healthcare even if they experience clinical symptoms.^90^ Multiple factors are likely to contribute to the variations in the prevalence/proportionate morbidity of travellers with STIs. It would be useful if future prospective studies focus on differentiating the factors that may influence the acquisition or the diagnosis of STIs among different travelling populations.

Although the prevalence/proportionate morbidity of travellers with STIs varied among the different groups, certain risk factors were found to be consistent among all travellers. These included behavioural factors (e.g. having multiple sexual partners, unprotected sexual intercourse, use of injectable drugs and having sex with sex workers) and demographic factors (e.g. older age, male, low education level, solo travellers). These risk factors are similar to those observed in the general population (non-travellers).^91^^,^^92^ This highlights the utility of using known risk factors for STI acquisition and transmission in the context of travel until more specific travel-related information is available. Well-established prevention and control measures for general populations might help to mitigate the transmission and impact of STIs among travellers. However, distinct travel-related risk factors for STIs were identified that supports the need to develop travel-specific prevention and control measures, e.g. risk factors such as short-term travel, Southeast Asian destination, VFR travel, not being vaccinated against HBV and lack of pre-travel advice more generally.

A short duration of travel (less than a month) was associated with an increased risk of STIs in the paper by Matteelli A and colleagues.^52^ This finding aligns with the US Centres for Disease Control and Prevention (CDC) report, which indicates that most travel-related infectious diseases occur within the initial month of travel.^93^ Caution is warranted when interpreting this data, as establishing a causal relationship between acquisition of STIs and shorter trip duration is challenging. The observed risk might simply be due to most travellers going on short trips, rather than any risk inherent in short trips. Consequently, the link between the duration of travel and the risk of STIs may be complex and multifactorial, depending on the risk behaviours of travellers, the nature of specific STIs and the different incubation periods of these infections. In contrast, a study by Crawford *et al*.^29^ revealed that extended stays at travel destinations could increase the high-risk sexual behaviours of travellers. One could postulate that spending an extended period in a travel destination may foster a sense of familiarity with the local community, culture and environment, potentially resulting in increased sexual activity with locals and consequent increase in acquisition of STIs. An investigation of international backpackers staying in Australia between 4 and 12 weeks reported that 55% of those who tested positive for *C. trachomatis* had engaged in sexual contact with ‘locals’ (i.e. people living permanently in Australia).^66^ However, existing evidence indicates that the impact of travel duration on the risk of STI acquisition varies. These findings highlight the complexity of developing safer sex messages and care plans for travellers.

The geographic region visited by travellers has been shown to play a significant role in the acquisition and transmission of STIs.^32^ The findings of our review demonstrate an increased risk of STIs among travellers returning from Southeast Asia. This finding is particularly concerning given that Southeast Asia has been identified as a region experiencing a high incidence of antimicrobial resistance, including the spread of drug-resistant STIs.^94^^,^^95^ A review conducted by Vicente *et al*.^22^ identified Southeast Asia as the geographical hotspot for the emergence and acquisition of drug-resistant gonorrhoea. The first global reports of multi-drug resistant STIs, notably gonorrhoea, were identified in Australia and the UK among travellers returning from Southeast Asia.^18^ Therefore, to reduce the transmission of STIs, it is crucial for practitioners providing care to travellers to design messages incorporating the geographic location of travel, as well as the travellers’ involvement in high-risk sexual activity in those regions.

Our review identified that compared to non VFR travellers, VFR travel is associated with an increased risk of STIs along with the known increased risk of other infectious diseases.^96^ Compounding this problem, <16% of VFRs travellers seek pre-travel consultation, compared to 62% in other groups of travellers (i.e. tourist travellers).^97^ This finding highlights the importance of investigating and targeting the unique factors that contribute to the vulnerability of VFRs to reduce the transmission and acquisition of STIs within this group.

Our review also found that travellers who sought pre-travel advice had a lower risk of STIs. Travellers who seek pre-travel advice likely differ from those who do not seek advice, i.e. the former may be more inclined to take preventive measures. Healthcare providers should be familiar with current STI prevention recommendations outlined by various studies and guidelines.^93^^,^^98^ These recommendations include ensuring current vaccinations against HBV, offering pre-exposure prophylaxis for HIV and encouraging consistent condom use. A comprehensive package of customized pre-travel advice might enhance awareness of STIs, modify high-risk sexual behaviours and mitigate the transmission of STIs. In particular, evidence has shown that consistent condom use reduces the incidence of STIs.^99^

In the current review, determining the timing of acquisition of an STI can be challenging, particularly for diseases with long incubation periods like HBV. The asymptomatic transmission of some STIs (e.g. syphilis, HIV and *C. trachomatis*) further complicates matters, along with the variable incubation periods of STIs. For example, gonorrhoea, HIV, syphilis (chancre) and *C. trachomatis* have short incubation periods (1–4 weeks);^100^ this may result in the first signs or symptoms of the disease appearing during the travel period or shortly after arrival home. In contrast, other STIs can have longer incubation times (HBV and human papillomavirus), resulting in symptoms not appearing until months or even years after acquisition.^100^^,^^101^ Some travellers will be unaware that they have acquired an STI during their travels. The long incubation periods of some STIs and the absence of baseline (prior to travelling) STI testing/screening makes it impossible to document a clear causal link between the travel and the infection. Additionally, only a limited number of studies (*n =* 7, 21.8%) reported the time between the onset of clinical symptoms and the date of return or clinic investigations. This further complicates the efforts to establish a definitive link and may contribute to discrepancies in the prevalence of travellers with STIs.

It is also worth noting that out of the 32 studies, 19 included testing/screening for symptomatic STIs in travellers. Given that the majority of STIs are asymptomatic,^102^ many travellers are unaware that they have an STI and may not seek medical attention in the absence of symptoms. This means the reported prevalence of travellers with STIs is likely underestimated. Moreover, our findings revealed higher proportionate morbidity of symptomatic VFRs travellers with STIs [e.g. HIV (0.72%) and syphilis (1.67%)], backpackers [*C. trachomatis* (6.58%)] and expatriates [unspecified STIs (4.76%)], while there was a higher prevalence of asymptomatic STIs among MSM travellers [HIV (26.0%), HBV (25.0%)] and business travellers [syphilis (0.74%)]. The overall findings of the review support the recommendations of the US CDC that travellers who engage in high-risk sexual activity during their trips should undergo post-travel counselling and STIs screening before engaging in sexual activity upon return.^93^

The most common STIs identified in our review varied by category of travellers. For instance, *C. trachomatis* was most common in backpackers; gonorrhoeae in MSM; syphilis in VFR; and HIV, HAV, HBV and LGV in MSM. Our findings somewhat align with a study conducted by Ansart S *et al.*,^103^ where the main STIs diagnosed among travellers returning from the tropics were gonorrhoea, herpes simplex virus 2, *C. trachomatis*, syphilis and HIV, whereas the study by Hawkes S *et al.*^71^ reported that among UK travellers returned from overseas, STIs identified included gonorrhoea, *C. trachomatis*, primary human papillomavirus, primary herpes simplex and trichomonas vaginalis. However, the studies are not fully comparable, as our study investigated the prevalence/proportionate morbidity of STIs categorized by type of travellers. In addition, the variations in reported spectrum of STIs between our study and other reports may be due to the inclusion criteria of our review, which focuses on common types of STIs and sexually transmissible BBVs (i.e. *C. trachomatis*, gonorrhoea, syphilis, trichomoniasis, Zika virus, HAV, LGV, HIV, HBV and HCV). The current review had several limitations. Firstly, it was impossible to estimate the destination-specific prevalence/proportionate morbidity of travellers with STIs, given that travel details such as trip destination and duration were unavailable in most studies. Secondly, travellers were tested while overseas or upon return and none of the studies had a pre-travel STIs test to rule out the possibility of STIs prior to international travel, precluding the STIs acquisition rate calculation. Thirdly, the generalizability of the findings may be limited, as more than two-third of the studies (68.42%) included international travellers from Europe and USA, and behavioural factors and risk profiles may differ for travellers from other regions (e.g. Asia, Latin America). Fourthly, establishing a causal link between travel and the acquisition of STIs is challenging due to absence of pre-travel testing and prolonged incubation period. Fifthly, we have included studies reporting syphilis among VFRs with suggestive signs of STIs or symptomatic travellers; however, diagnosing syphilis cases among VFRs is challenging. The non-specific nature of clinical presentations for STIs/concurrent symptoms of multiple STIs, combined with the inability of the serologic tests to differentiate venereal syphilis to other non-venereal treponematoses, makes it difficult to ascertain the timing of syphilis infection and the proportionate morbidity of syphilis within this population. Finally, the longer a traveller stays in a particular area (e.g. long-term travellers or expatriates), the less likely they are to present to a medical facility and be asked about their travel history, potentially leading to inadequate information capture. Thus, the study was restricted to travellers with stays of up to 1 year.

In conclusion, the prevalence/proportionate morbidity of travellers with STIs varied among different types of travellers. The risk factors for contracting an STI were found to be VFRs, short-term travel, lack of pre-travel advice and not being vaccinated against HBV. There are significant implications for travel medicine, highlighting the need for targeted interventions to mitigate travel-related STIs for all traveller groups. Healthcare providers need tailored prevention strategies to reduce the risk of STIs among travellers, especially those at higher risk. Additionally, travellers need to be encouraged to attend pre-travel consultations and accept appropriate vaccinations to help reduce their risk of STIs. Lastly, further research is needed to validate the effectiveness of pre-travel advice in among travellers. Investigation of effective, evidence-based strategies to encourage travellers to practice safer sex, and reducing STIs acquisition rates should be a priority.

## Funding

The authors did not receive funds for conducting the review. However, W.S. was granted the University of Queensland Research Training Stipend Scholarship to pursue his PhD. C.L.L. was supported by an NHMRC Investigator Grant (APP1193826).

## Author contributions

Wondimeneh Shiferaw (Conceptualization, Investigation, Methodology, Visualization, Writing—original draft, Writing—review & editing [equal]), Beatris Martin (Investigation, Writing—review & editing [equal]), Judith A. Dean (Supervision, Writing—review & editing [equal]), Deborah Mills (Supervision, Writing—review & editing [equal]), Colleen Lau (Supervision, Writing—review & editing [equal]), David Paterson (Supervision, Writing—review & editing [equal]), Kenneth Koh (Writing—review & editing [equal]), Lars Eriksson (Resources, Writing—review & editing [equal]) and Luis Furuya-Kanamori (Conceptualization, Supervision, Writing—review & editing [equal])

## Conflict of interest

None declared.

## Data availability

The data underlying this article are available in the article and supplementary materials.

## Supplementary Material

Supplementary_material_taae038
