# Supplementary material for: A systematic review and meta-analysis of sexually transmitted infections and blood-borne viruses in travellers
Source: J Travel Med. 2024 Mar 4;31(4):taae038. doi: 10.1093/jtm/taae038 (PMC11149723; doi:10.1093/jtm/taae038)
Supplement: Supplementary_material_taae038 [file supplementary_material_taae038.docx]

**Supplementary material**

S1. The PRISMA checklist for reporting the review.

| **Section and Topic** | **Item #** | **Checklist item** | **Location where item is reported** |
| --- | --- | --- | --- |
| **TITLE** | | |  |
| Title | 1 | Identify the report as a systematic review. | #1 |
| **ABSTRACT** | | |  |
| Abstract | 2 | See the PRISMA 2020 for Abstracts checklist. | #1 |
| **INTRODUCTION** | | |  |
| Rationale | 3 | Describe the rationale for the review in the context of existing knowledge. | #3-4 |
| Objectives | 4 | Provide an explicit statement of the objective(s) or question(s) the review addresses. | #4 |
| **METHODS** | | |  |
| Eligibility criteria | 5 | Specify the inclusion and exclusion criteria for the review and how studies were grouped for the syntheses. | #6 |
| Information sources | 6 | Specify all databases, registers, websites, organisations, reference lists and other sources searched or consulted to identify studies. Specify the date when each source was last searched or consulted. | #5 |
| Search strategy | 7 | Present the full search strategies for all databases, registers, and websites, including any filters and limits used. | #5 and S2 |
| Selection process | 8 | Specify the methods used to decide whether a study met the inclusion criteria of the review, including how many reviewers screened each record and each report retrieved, whether they worked independently, and if applicable, details of automation tools used in the process. | #5-6 |
| Data collection process | 9 | Specify the methods used to collect data from reports, including how many reviewers collected data from each report, whether they worked independently, any processes for obtaining or confirming data from study investigators, and if applicable, details of automation tools used in the process. | #5-6 |
| Data items | 10a | List and define all outcomes for which data were sought. Specify whether all results that were compatible with each outcome domain in each study were sought (e.g. for all measures, time points, analyses), and if not, the methods used to decide which results to collect. | #6 |
|  | 10b | List and define all other variables for which data were sought (e.g. participant and intervention characteristics, funding sources). Describe any assumptions made about any missing or unclear information. | #7 |
| Study risk of bias assessment | 11 | Specify the methods used to assess risk of bias in the included studies, including details of the tool(s) used, how many reviewers assessed each study and whether they worked independently, and if applicable, details of automation tools used in the process. | #7 |
| Effect measures | 12 | Specify for each outcome the effect measure(s) (e.g. risk ratio, mean difference) used in the synthesis or presentation of results. | #8 |
| Synthesis methods | 13a | Describe the processes used to decide which studies were eligible for each synthesis (e.g. tabulating the study intervention characteristics and comparing against the planned groups for each synthesis (item #5)). | #8 |
|  | 13b | Describe any methods required to prepare the data for presentation or synthesis, such as handling of missing summary statistics, or data conversions. | #7-8 |
|  | 13c | Describe any methods used to tabulate or visually display results of individual studies and syntheses. | #8 |
|  | 13d | Describe any methods used to synthesize results and provide a rationale for the choice(s). If meta-analysis was performed, describe the model(s), method(s) to identify the presence and extent of statistical heterogeneity, and software package(s) used. | #8 |
|  | 13e | Describe any methods used to explore possible causes of heterogeneity among study results (e.g. subgroup analysis, meta-regression). | NA |
|  | 13f | Describe any sensitivity analyses conducted to assess robustness of the synthesized results. | NA |
| Reporting bias assessment | 14 | Describe any methods used to assess risk of bias due to missing results in a synthesis (arising from reporting biases). | NA |
| Certainty assessment | 15 | Describe any methods used to assess certainty (or confidence) in the body of evidence for an outcome. | #8 |
| **RESULTS** | | |  |
| Study selection | 16a | Describe the results of the search and selection process, from the number of records identified in the search to the number of studies included in the review, ideally using a flow diagram. | #9 |
|  | 16b | Cite studies that might appear to meet the inclusion criteria, but which were excluded, and explain why they were excluded. | #9 |
| Study characteristics | 17 | Cite each included study and present its characteristics. | #9 |
| Risk of bias in studies | 18 | Present assessments of risk of bias for each included study. | #13 |
| Results of individual studies | 19 | For all outcomes, present, for each study: (a) summary statistics for each group (where appropriate) and (b) an effect estimate and its precision (e.g. confidence/credible interval), ideally using structured tables or plots. | #10-13 |
| Results of syntheses | 20a | For each synthesis, briefly summarise the characteristics and risk of bias among contributing studies. | #10-12 |
|  | 20b | Present results of all statistical syntheses conducted. If meta-analysis was done, present for each the summary estimate and its precision (e.g. confidence/credible interval) and measures of statistical heterogeneity. If comparing groups, describe the direction of the effect. | 11-13 and S3 |
|  | 20c | Present results of all investigations of possible causes of heterogeneity among study results. | NA |
|  | 20d | Present results of all sensitivity analyses conducted to assess the robustness of the synthesized results. | NA |
| Reporting biases | 21 | Present assessments of risk of bias due to missing results (arising from reporting biases) for each synthesis assessed. | NA |
| Certainty of evidence | 22 | Present assessments of certainty (or confidence) in the body of evidence for each outcome assessed. | 11-13 |
| **DISCUSSION** | | |  |
| Discussion | 23a | Provide a general interpretation of the results in the context of other evidence. | #13 |
|  | 23b | Discuss any limitations of the evidence included in the review. | #17 |
|  | 23c | Discuss any limitations of the review processes used. | #17 |
|  | 23d | Discuss implications of the results for practice, policy, and future research. | #13-17 |
| **OTHER INFORMATION** | | |  |
| Registration and protocol | 24a | Provide registration information for the review, including register name and registration number, or state that the review was not registered. | PROSPERO Number: CRD42023388857 |
|  | 24b | Indicate where the review protocol can be accessed, or state that a protocol was not prepared. | Prospero |
|  | 24c | Describe and explain any amendments to information provided at registration or in the protocol. | #5 |
| Support | 25 | Describe sources of financial or non-financial support for the review, and the role of the funders or sponsors in the review. | #18 |
| Competing interests | 26 | Declare any competing interests of review authors. | #18 |
| Availability of data, code and other materials | 27 | Report which of the following are publicly available and where they can be found: template data collection forms; data extracted from included studies; data used for all analyses; analytic code; any other materials used in the review. | #18 |

**S2**. Search strategy for the different databases

***PubMed***

(("Sexually Transmitted Diseases"[Mesh:Noexp] OR "Sexually Transmitted Diseases, Bacterial"[ Mesh:Noexp] OR "Sexually Transmitted Diseases, Viral"[Mesh:Noexp] OR "Sexually Transmitted Disease*"[tiab] OR STI[tiab] OR STD[tiab] OR "gonorrhea"[MeSH Terms] OR "gonorrhea"[tiab] OR "hepatitis a"[MeSH Terms] OR "hepatitis a"[tiab] OR " zika virus "[MeSH Terms] OR "zika virus"[tiab] OR "Lymphogranuloma venereum"[MeSH Terms] OR "Lymphogranuloma venereum"[tiab] OR “mpox "[MeSH Terms] OR "mpox"[tiab] OR “hepatitis b"[MeSH Terms] OR "hepatitis b"[tiab] OR "syphilis"[MeSH Terms] OR "syphilis"[tiab] OR "chlamydia"[MeSH Terms] OR "chlamydia"[tiab] OR "Chlamydia Infections"[Mesh] OR "chancroid"[MeSH Terms] OR "chancroid"[tiab] OR HIV [MeSH Terms] OR "AIDS"[tiab]) AND ("Travel Medicine"[Mesh] OR "Transients and Migrants"[Mesh] OR "Emigrants and Immigrants"[Mesh] OR "Emigration and Immigration"[Mesh] OR "Travel"[Mesh] OR "Tourism"[Mesh] OR expat*[tiab] OR migrant*[tiab] OR traveler*[tiab] OR traveller*[tiab] OR touris*[tiab] OR travel*[tiab] OR (travel*[tiab] OR visit*[tiab] AND (holiday*[tiab] OR overseas*[tiab] OR abroad*[tiab] OR foreign*[tiab])))) AND ("Epidemiology"[Mesh] OR "epidemiology"[Subheading] OR "epidemiolog*"[tiab] OR "surveill*"[tiab] OR Prevalence[mesh] OR prevalen*[tiab] OR endemic[tiab] OR monitor*[ti] OR proportion[tiab] OR screen*[tiab] OR "Mass Screening"[Mesh] OR incidence[tiab])

***Embase***

('sexually transmitted disease'/exp OR 'sexually transmitted disease*':ti,ab OR sti:ti,ab OR std:ti,ab OR 'gonorrhea'/exp OR gonorrhea:ti,ab OR 'syphilis'/exp OR syphilis:ti,ab OR 'chlamydia'/exp OR chlamydia:ti,ab OR 'chlamydia infection'/exp OR 'hepatitis a'/exp OR 'hepatitis a':ti,ab OR 'zika virus'/exp OR 'zika':ti,ab OR ' Lymphogranuloma venereum /exp OR Lymphogranuloma venereum':ti,ab OR 'hepatitis b'/exp OR 'hepatitis b':ti,ab OR 'Mpox'/exp OR 'mpox':ti,ab OR 'ulcus molle'/exp OR chancroid:ti,ab) AND ('emporiatrics'/exp OR 'migration'/exp OR 'travel'/exp OR 'tourism'/exp OR expat*:ti,ab OR 'migrant'/exp OR migrant*:ti,ab OR traveler*:ti,ab OR traveller*:ti,ab OR touris*:ti,ab OR travel*:ti,ab OR ((travel*:ti,ab OR visit*:ti,ab) AND (holiday*:ti,ab OR overseas*:ti,ab OR abroad*:ti,ab OR foreign*:ti,ab))) AND ('epidemiology'/exp OR 'epidemiology' OR epidemiolog*:ti,ab OR surveill*:ti,ab OR 'prevalence'/exp OR prevalen*:ti,ab OR endemic:ti,ab OR monitor*:ti OR proportion:ti,ab OR screen*:ti,ab OR 'mass screening'/exp OR 'screening'/exp OR incidence:ti,ab) AND [embase]/lim AND ([article]/lim OR [article in press]/lim OR [review]/lim) AND ([article]/lim OR [article in press]/lim OR [review]/lim) AND [embase]/lim

***CINAHL via EBSCOhost***

( ((MH "Sexually Transmitted Diseases") OR MH "Sexually Transmitted Diseases, Bacterial+" OR (MH "Sexually Transmitted Diseases, Viral") OR (TI "Sexually Transmitted Disease*" OR AB "Sexually Transmitted Disease*") OR (TI STI OR AB STI) OR (TI STD OR AB STD) OR (MH gonorrhea+) OR (TI gonorrhea OR AB gonorrhea) OR (MH hepatitis a+) OR (TI hepatitis a OR AB hepatitis a) OR (MH zika virus +) OR (TI zika virus OR AB zika virus) OR (MH Lymphogranuloma venereum +) OR (TI Lymphogranuloma venereum OR AB Lymphogranuloma venereum) OR OR (MH mpox +) OR (TI mpox OR AB mpox) OR (MH hepatitis b+) OR (TI hepatitis b OR AB hepatitis b) OR (MH syphilis+) OR (TI syphilis OR AB syphilis) OR (MH chlamydia+) OR (TI chlamydia OR AB chlamydia) OR (MH "Chlamydia Infections+") OR (MH chancroid+) OR (TI chancroid OR AB chancroid) OR (MH HIV/AIDS+) OR (TI HIV/AIDS OR AB HIV/AIDS)) ) AND ( (MH "Travel Health") OR (MH "Transients and Migrants") OR (MH "Emigration and Immigration") OR (MH Travel+) OR (MH Tourism+) OR (TI expat* OR AB expat*) OR (TI migrant* OR AB migrant*) OR (TI traveler* OR AB traveler*) OR (TI traveller* OR AB traveller*) OR (TI touris* OR AB touris*) OR (TI travel* OR AB travel*) OR ((TI travel* OR AB travel*) OR (TI visit* OR AB visit*) AND ((TI holiday* OR AB holiday*) OR (TI overseas* OR AB overseas*) OR (TI abroad* OR AB abroad*) OR (TI foreign* OR AB foreign*))) ) AND ( ((MH Epidemiology+) OR "Epidemiology" OR (TI epidemiolog* OR AB epidemiolog*) OR (TI surveill* OR AB surveill*) OR (MH Prevalence) OR (TI prevalen* OR AB prevalen*) OR (TI endemic OR AB endemic) OR (TI monitor*) OR (TI proportion OR AB proportion) OR (TI screen* OR AB screen*) OR (MH "Health Screening+") OR (TI incidence OR AB incidence)) )

***Cochrane Library***

ID Search

#1 [mh ^"Sexually Transmitted Diseases"] OR "Sexually Transmitted Diseases, Bacterial[ Mesh:Noexp]" OR [mh ^"Sexually Transmitted Diseases, Viral"] OR ("Sexually Transmitted" NEXT Disease*):ti,ab OR STI:ti,ab OR STD:ti,ab OR [mh gonorrhea] OR gonorrhea:ti,ab OR [mh hepatitis a] OR hepatitis a:ti,ab OR [mh zika virus] OR zika virus:ti,ab OR [mh Lymphogranuloma venereum] OR Lymphogranuloma venereum:ti,ab OR [mh mpox] OR mpox a:ti,ab OR [mh hepatitis b] OR hepatitis b:ti,ab OR [mh syphilis] OR syphilis:ti,ab OR [mh chlamydia] OR chlamydia:ti,ab OR [mh "Chlamydia Infections"] OR [mh chancroid] OR chancroid:ti,ab OR [mh AIDS] OR HIV:ti,ab

#2 [mh "Travel Medicine"] OR [mh "Transients and Migrants"] OR [mh "Emigration and Immigration"] OR [mh Travel] OR [mh Tourism] OR expat*:ti,ab OR migrant*:ti,ab OR traveler*:ti,ab OR traveller*:ti,ab OR touris*:ti,ab OR travel*:ti,ab OR (travel*:ti,ab OR visit*:ti,ab AND (holiday*:ti,ab OR overseas*:ti,ab OR abroad*:ti,ab OR foreign*:ti,ab))

#3 [mh Epidemiology] OR [mh /EP] OR epidemiolog*:ti,ab OR surveill*:ti,ab OR [mh Prevalence] OR prevalen*:ti,ab OR endemic:ti,ab OR monitor*:ti OR proportion:ti,ab OR screen*:ti,ab OR [mh "Mass Screening"] OR incidence:ti,ab

#4 #1 AND #2 AND #3

***Web of Science Clarivate***

((TS=("Sexually Transmitted Diseases" OR "Sexually Transmitted Disease*" OR STI OR STD OR gonorrhea OR gonorrhea OR hepatitis a OR zika virus OR Lymphogranuloma venereum OR mpox OR hepatitis b OR syphilis OR chlamydia OR "Chlamydia Infections" OR chancroid OR "HIV/AIDS")) AND TS=("Travel Medicine" OR "Transients and Migrants" OR "Emigration and Immigration" OR Travel OR Tourism OR expat* OR migrant* OR traveler* OR traveller* OR touris* OR travel* OR (travel* OR visit* AND (holiday* OR overseas* OR abroad* OR foreign*)))) AND TS=(epidemiolog* OR surveill* OR Prevalence OR prevalen* OR endemic OR monitor* OR proportion OR screen* OR "Mass Screening" OR incidence)

***Scopus***

( INDEXTERMS ( epidemiology )  OR  "Epidemiology"  OR  TITLE-ABS ( epidemiolog* )  OR  TITLE-ABS ( surveill* )  OR  INDEXTERMS ( prevalence )  OR  TITLE-ABS ( prevalen* )  OR  TITLE-ABS ( endemic )  OR  TITLE ( monitor* )  OR  TITLE-ABS ( proportion )  OR  TITLE-ABS ( screen* )  OR  INDEXTERMS ( "Mass Screening" )  OR  TITLE-ABS ( incidence ) )  AND  ( INDEXTERMS ( "Travel Medicine" )  OR  INDEXTERMS ( "Transients and Migrants" )  OR  INDEXTERMS ( "Emigration and Immigration" )  OR  INDEXTERMS ( travel )  OR  INDEXTERMS ( tourism )  OR  TITLE-ABS ( expat* )  OR  TITLE-ABS ( migrant* )  OR  TITLE-ABS ( traveler* )  OR  TITLE-ABS ( traveller* )  OR  TITLE-ABS ( touris* )  OR  TITLE-ABS ( travel* )  OR  ( TITLE-ABS ( travel* )  OR  TITLE-ABS ( visit* )  AND  ( TITLE-ABS ( holiday* )  OR  TITLE-ABS ( overseas* )  OR  TITLE-ABS ( abroad* )  OR  TITLE-ABS ( foreign* ) ) ) )  AND  ( INDEXTERMS ( "Sexually Transmitted Diseases" )  OR  "Sexually Transmitted Diseases, Bacterial[ Mesh:Noexp]"  OR  INDEXTERMS ( "Sexually Transmitted Diseases, Viral" )  OR  TITLE-ABS ( "Sexually Transmitted Disease*" )  OR  TITLE-ABS ( sti )  OR  TITLE-ABS ( std )  OR  INDEXTERMS ( gonorrhea )  OR  TITLE-ABS ( gonorrhea ) OR INDEXTERMS (hepatitis a ) OR TITLE-ABS ( hepatitis a ) OR INDEXTERMS (zika virus) OR TITLE-ABS ( zika virus) OR INDEXTERMS (Lymphogranuloma venereum) OR TITLE-ABS ( Lymphogranuloma venereum) OR INDEXTERMS (mpox) OR TITLE-ABS ( mpox) OR INDEXTERMS (hepatitis b) OR TITLE-ABS ( hepatitis b) OR INDEXTERMS ( syphilis )  OR  TITLE-ABS ( syphilis )  OR  INDEXTERMS ( chlamydia )  OR  TITLE-ABS ( chlamydia )  OR  INDEXTERMS ( "Chlamydia Infections" )  OR  INDEXTERMS ( chancroid )  OR  TITLE-ABS ( chancroid ) )  AND  ( LIMIT-TO ( DOCTYPE ,  "ar" )  OR  LIMIT-TO ( DOCTYPE ,  "re" ) )

**S3.** Forest plots for the prevalence/proportionate morbidity of symptomatic and asymptomatic travellers with STIs by type of travellers

|  | Symptomatic patients [proportionate morbidity] | Asymptomatic patients [Prevalence] |
| --- | --- | --- |
| Tourists |  |  |
| Students |  |  |
| Business travellers |  |  |
| VFRs |  |  |
| Backpackers |  | 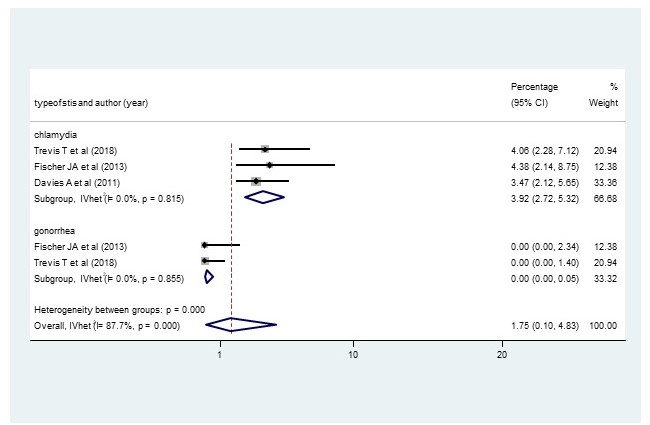 |
| Expatriates |  |  |
| International truck drivers | ---- |  |
| MSMs |  |  |

**S4.** Summary of the risk of bias assessments of the included studies.


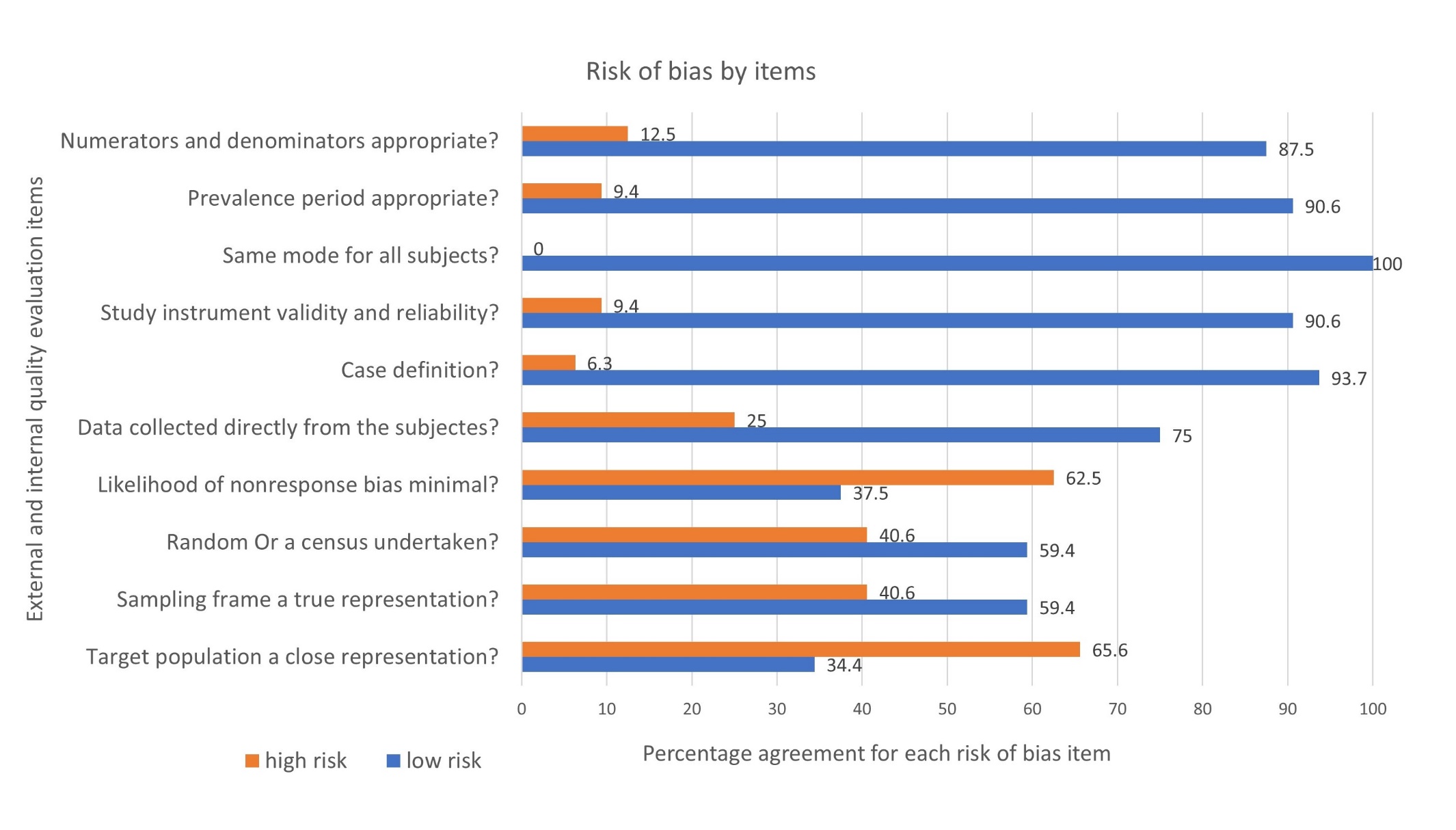


**S5.** Risk of bias assessments of the included individual studies.

| S.no | Author year | External Validity | | | | Internal Validity | | | | | |
| --- | --- | --- | --- | --- | --- | --- | --- | --- | --- | --- | --- |
|  |  | Target population a close representation | Sampling frame a true or close representation | Random selection? Or a census undertaken | Likelihood of nonresponse bias minimal | Data collected directly from the subjects | Case definition | Instrument validity and reliability | Same mode for all subjects | Length of prevalence period | Numerators and denominators appropriate |
|  | Alzahrani et al, 2009 | No | Yes | Yes | No | Yes | Yes | Yes | Yes | Yes | Yes |
|  | Angelo et al, 2018 | No | No | Yes | Yes | No | No | Yes | Yes | Yes | Yes |
|  | Angelo et al, 2020 | No | No | No | No | Yes | Yes | Yes | Yes | Yes | No |
|  | Ansart et al, 2005 | No | No | Yes | Yes | Yes | Yes | Yes | Yes | Yes | Yes |
|  | Beauté et al, 2017 | Yes | Yes | Yes | Yes | Yes | Yes | Yes | Yes | Yes | Yes |
|  | Bogglid et al, 2018 | Yes | Yes | No | No | Yes | Yes | Yes | Yes | Yes | Yes |
|  | Bonneux et al, 1988 | No | Yes | Yes | No | Yes | Yes | Yes | Yes | Yes | Yes |
|  | Botros et al, 2009 | Yes | Yes | No | No | Yes | Yes | Yes | Yes | Yes | No |
|  | Chimungu et al, 2020 | No | Yes | Yes | No | Yes | Yes | Yes | Yes | Yes | Yes |
|  | Davies et al, 2011 | No | Yes | No | No | Yes | No | Yes | Yes | No | Yes |
|  | Decraene et al, 2018 | Yes | Yes | Yes | No | Yes | Yes | Yes | Yes | Yes | Yes |
|  | Donachie et al, 2018 | No | No | No | No | Yes | Yes | Yes | Yes | Yes | Yes |
|  | Fang et al, 2018 | Yes | Yes | Yes | Yes | Yes | Yes | Yes | Yes | Yes | Yes |
|  | Field et al, 2010 | No | No | Yes | No | No | Yes | Yes | Yes | Yes | Yes |
|  | Fischer et al, 2015 | No | Yes | Yes | No | Yes | Yes | No | Yes | Yes | Yes |
|  | Han et al, 2019 | Yes | Yes | Yes | Yes | No | Yes | Yes | Yes | Yes | Yes |
|  | Hawkes et al, 1994 | No | Yes | Yes | Yes | Yes | Yes | Yes | Yes | No | Yes |
|  | Hawkes et al, 1995 | No | Yes | Yes | Yes | Yes | Yes | Yes | Yes | Yes | Yes |
|  | Kramer et al, 2008 | No | No | No | No | Yes | Yes | Yes | Yes | Yes | No |
|  | Matteelli et al, 2013 | No | No | Yes | No | No | Yes | No | Yes | Yes | Yes |
|  | McNulty et al 2018 | No | Yes | Yes | No | No | Yes | No | Yes | No | Yes |
|  | Nbundi et al, 2018 | No | No | No | No | Yes | Yes | Yes | Yes | Yes | Yes |
|  | Petridou et al, 2019 | No | No | No | No | Yes | Yes | Yes | Yes | Yes | Yes |
|  | Porse et al, 2018 | No | No | No | No | Yes | Yes | Yes | Yes | Yes | Yes |
|  | Schlagenhauf et al, 2015 | Yes | Yes | Yes | Yes | Yes | Yes | Yes | Yes | Yes | Yes |
|  | Steffen et al, 1987 | Yes | Yes | No | Yes | Yes | Yes | Yes | Yes | Yes | Yes |
|  | Struve et al, 1995 | Yes | No | Yes | Yes | No | Yes | Yes | Yes | Yes | No |
|  | Trevis et al, 2018 | No | No | No | No | Yes | Yes | Yes | Yes | Yes | Yes |
|  | Truoung et al, 2018 | No | Yes | No | No | Yes | Yes | Yes | Yes | Yes | Yes |
|  | Wilson, 2014 | No | No | No | No | Yes | Yes | Yes | Yes | Yes | Yes |
|  | Wu et al, 2020 | Yes | Yes | Yes | Yes | No | Yes | Yes | Yes | Yes | Yes |
|  | Zoldi et al, 2018 | Yes | Yes | Yes | Yes | No | Yes | Yes | Yes | Yes | Yes |
